# DERBI: A Digital Method to Help Researchers Offer “Right-to-Know” Personal Exposure Results

**DOI:** 10.1289/EHP702

**Published:** 2017-02-01

**Authors:** Katherine E. Boronow, Herbert P. Susmann, Krzysztof Z. Gajos, Ruthann A. Rudel, Kenneth C. Arnold, Phil Brown, Rachel Morello-Frosch, Laurie Havas, Julia Green Brody

**Affiliations:** 1 Silent Spring Institute, Newton, Massachusetts, USA; 2 Harvard John A. Paulson School of Engineering and Applied Sciences, Cambridge, Massachusetts, USA; 3 Social Science Environmental Health Research Institute, Northeastern University, Boston, Massachusetts, USA; 4 Department of Environmental Science, Policy and Management and School of Public Health, University of California, Berkeley, California, USA; 5 Child Health and Development Studies Participant Advisory Council, Berkeley, California, USA

## Abstract

Researchers and clinicians in environmental health and medicine increasingly show respect for participants and patients by involving them in decision-making. In this context, the return of personal results to study participants is becoming ethical best practice, and many participants now expect to see their data. However, researchers often lack the time and expertise required for report-back, especially as studies measure greater numbers of analytes, including many without clear health guidelines. In this article, our goal is to demonstrate how a prototype digital method, the Digital Exposure Report-Back Interface (DERBI), can reduce practical barriers to high-quality report-back. DERBI uses decision rules to automate the production of personalized summaries of notable results and generates graphs of individual results with comparisons to the study group and benchmark populations. Reports discuss potential sources of chemical exposure, what is known and unknown about health effects, strategies for exposure reduction, and study-wide findings. Researcher tools promote discovery by drawing attention to patterns of high exposure and offer novel ways to increase participant engagement. DERBI reports have been field tested in two studies. Digital methods like DERBI reduce practical barriers to report-back thus enabling researchers to meet their ethical obligations and participants to get knowledge they can use to make informed choices.

## Introduction

With technological advances in personal exposure measurement for environmental chemicals—such as testing of blood, urine, breast milk, and household dust and air—scientists can detect a wide array of emerging contaminants, often at very low levels. These high-resolution data provide crucial opportunities for research, but the large data sets pose challenges for reporting results to study participants. Detection capabilities outpace clear health guidelines, so distilling messages that interpret results is difficult even for experts, and, in studies with large numbers of chemicals, reports need to be individually tailored to focus participants on the most relevant information.

In the past, environmental health researchers avoided these problems by reporting only individual results that were above a clinical health guideline, such as a blood lead above the reference level set by the Centers for Disease Control and Prevention (CDC 2016). However, new practices for personal report-back have emerged over the past 15 years as participants in community-based participatory research (CBPR) studies have requested to learn all of their results. Because report-back fulfills core CBPR goals of partnership, empowerment, and co-learning (Minkler and Wallerstein 2008), researchers supported participants’ right to know—and act—on personal results (Brody et al. 2007; Morello-Frosch et al. 2009; Quandt et al. 2004). In the language of research ethics, report-back promotes autonomy, justice, and beneficence, while balancing concerns about potential harm from reporting data of uncertain consequence (Brody et al. 2007).

Influenced by CBPR and trends in genetics and medicine (Esch et al. 2016; Hood and Auffray 2013; Morello-Frosch et al. 2015; Wolf 2013), broader institutional attitudes are also shifting toward respect for autonomy and recognition of report-back as an ethical best practice. Several U.S. expert reports, such as those by the National Academy of Sciences (NRC 2006), the Boston University Consensus Conference (Nelson et al. 2009), and the U.S. Environmental Protection Agency (U.S. EPA 2008), recommend report-back, and the California Biomonitoring Program is required by law to make individual results available (State of California 2006). Updates to federal regulations in 2014 strengthened the rights of patients to access laboratory test results (CMS, CDC, OCR 2014), and one of the key principles of the President’s Precision Medicine Initiative is the responsible return of results (The White House 2015). Large-scale biomonitoring efforts in Canada and Europe also require report-back (Becker et al. 2014; Day et al. 2007).

The ethical rationale for report-back is supported by research showing that report-back can benefit both participants and researchers. In a study that surveyed its participants and in studies that offer report-back as an option in informed consent protocols, 90% or more of participants wish to receive their results (e.g., Adams et al. 2011; Altman et al. 2008; Barlow and Kushi 2011). Interviews reveal that participants are not excessively worried; rather, many are prompted to think about personal sources of exposure and motivated to take action (Brody et al. 2014). While different levels of evidence are available for different chemicals, there is emerging scientific consensus about the potential for harmful health effects and for exposure reduction for many biomonitored chemicals (Bennett et al. 2016; Di Renzo et al. 2015; Gore et al. 2015; UNEP/WHO 2013). Report-back empowers participants to learn about the science and consider precautionary action and public policies, and it promotes justice by reducing disparities in access to knowledge (Morello-Frosch et al. 2009). Researchers who report back benefit by building trust with participants (Hernick et al. 2011), which improves study recruitment and retention and opens the door for investigating the behaviors or products underlying unusual results (Brody et al. 2014).

Still, the rate of report-back among academic researchers remains low, and the major U.S. biomonitoring effort, the National Health and Nutrition Examination Survey (NHANES), which tests for about 200 environmental chemicals, reports only levels for seven metals (CDC 2015). Researchers and institutional review boards (IRBs) cite concerns about causing anxiety, ensuring privacy, and developing meaningful content in the face of scientific uncertainty (Brody et al. 2014; Morello-Frosch et al. 2009), and these are important topics for further study. However, best practices help mitigate these issues (Dunagan et al. 2013), and a growing number of studies have successfully navigated these challenges in their report-back (Adams et al. 2011; Altman et al. 2008; Haynes et al. 2016; Hernick et al. 2011; Judge et al. 2016; Wu et al. 2009).

At the same time, researchers also cite practical obstacles including lack of time, funding, and expertise (Brody et al. 2014). For example, the time needed to individually personalize reports to highlight notable findings—essential to helping participants understand their results—quickly scales with study enrollment, and providing constructive context requires transdisciplinary knowledge and communications skills.

We believe that digital methods are key to overcoming these practical barriers and can help bring research practices into line with modern ethics, but we are not aware of any other environmental health studies that have leveraged these methods. To help advance solutions, we introduce the Digital Exposure Report-Back Interface (DERBI) as a prototype software framework for generating personal exposure reports for print or the web. DERBI also includes integrated data visualization tools to help researchers analyze individual and study-wide patterns of exposure. We designed DERBI to meet three core objectives: *a*) to produce user-centered reports that are understandable and useful to a wide range of audiences, *b*) to provide a scalable platform for report-back for studies of all sizes, and *c*) to deliver value-added research tools for investigators that enhance data exploration and create new opportunities for data collection. This communication describes our approach and invites discussion, improvements, and further experimentation.

### Collaborations to Develop DERBI

We developed DERBI to fill a need for efficient report-back tools in our own research and that of others. Since 1999, four of the authors of this article (J.B., P.B., R.M-F., and R.R.) have conducted environmental exposure studies that reported results to several hundred participants for more than 100 chemicals. In the early studies, the investigators—drawing on their expertise in psychology, sociology, environmental public health, and toxicology—manually developed reports and systematically evaluated participants’ experience with report-back. To broaden their investigation of report-back, the same authors developed the Personal Exposure Report-Back Ethics (PERE) Study, supported by the National Institute of Environmental Health Sciences (NIEHS). The PERE Study completed 193 semi-structured, hour-long interviews with researchers, IRB members, and participants in nine other environmental exposure studies that included personal exposure report-back (Brody et al. 2014; Judge et al. 2016; Ramirez-Andreotta et al. 2016). Analyses are ongoing, but as the interviews—and the authors’ experience—revealed practical barriers to report-back, we conceived of digital methods as a solution for efficiently giving participants personally relevant information in large studies.

With additional NIEHS support, we developed new collaborations to build and test DERBI. To gain expertise in digital interface design, we partnered with computer scientists K. Gajos and K. Arnold in the Intelligent Interactive Systems Group at Harvard Paulson School of Engineering and Applied Sciences. We worked with two studies to refine and field test DERBI with culturally and economically diverse participants: the CDC Green Housing Study (GHS) and the Child Health and Development Studies (CHDS).

In GHS, researchers used DERBI to produce hard copy reports for 94 households living in Boston and Cincinnati public housing, including many immigrant families. GHS is a study of children with asthma living in newly renovated public housing (Coombs et al. 2016). Children were tested for asthma-related health indicators, and their urine samples and household air and dust were tested for selected pesticides, flame retardants, fragrance chemicals, combustion byproducts, polychlorinated biphenyls (PCBs), phthalates, and parabens and other personal care chemicals. Parents received reports for their children at a community meeting.

In its second deployment, researchers used DERBI in CHDS, a multi-generation cohort study that originated with women enrolled during their pregnancies in Oakland, California, in 1959–1967 (CHDS 2016). The CHDS used DERBI to create and deliver web reports for 295 women in the second generation. Measurements included recent blood levels of organochlorine pesticides, brominated flame retardants, perfluoroalkyl and polyfluoroalkyl substances (PFASs), PCBs, and lipids. Of the women who participated, 50% were African American. Participants completed interviews about their experience before and after receiving their reports.

The GHS and CHDS investigators chose to participate because our team provided access to experience in report-back methods and efficient implementation using DERBI. User testing, results reporting, and interview protocols were approved by IRBs at CHDS, CDC, and at Harvard University and Northeastern University. Informed consent was obtained before user testing, report-back, and interviews. The CHDS participants also consented to having web analytic data collected while viewing their reports.

## Discussion

Digital technology can automate many of the time-intensive processes associated with producing personalized reports. DERBI creates user-centered reports that can be customized at the individual and study-wide levels, while leveraging digital methods that minimize researcher effort. Intended for release as open-source software, our goal is for DERBI to be a model for how digital tools can streamline report-back in exposure biomonitoring studies or any study collecting personal data on individuals.

### User-Centered Design

Participants in biomonitoring studies—regardless of their formal education—often share the experience of learning that contaminants they never heard of were detected in their body or home. However, individual and cultural differences in literacy, numeracy, information-seeking style, and motivation affect what people want to know about their results and how they respond to different presentations of data. User-centered design—an iterated process that focuses on addressing the needs and limitations of a product’s end users—provides a strategy to accommodate as many of these differences as possible within a single design (Holtzblatt 2009).

**Figure 1:**
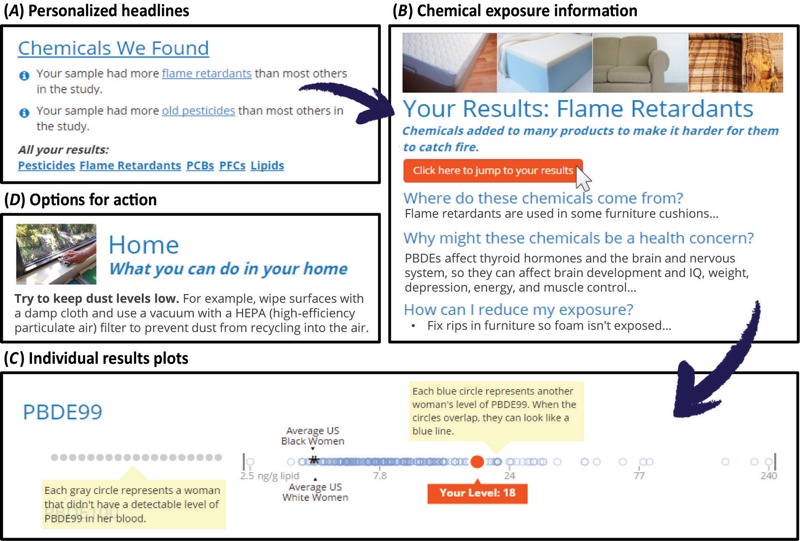
(*A*) During a typical user experience, participants first see their notable exposure results (headlines) on the Summary page. (*B*) Clicking on a chemical name takes them to more information about that chemical group, including exposure sources, health effects, options for taking action, and complete results. Chemical pages include personalized graphs that depict a participant’s chemical level in the context of the study distribution. (*C*) Digital features like explanatory pop-ups help people interpret the information-rich plots. (*D*) Participants can find more tips for reducing their exposure in the “What You Can Do” section. Other sections of the report include “Health Concerns” and “Overall Study Results” (not shown). To view an example web-based Digital Exposure Report-Back Interface (DERBI) report, see http://silentspring.org/research-area/digital-exposure-report-back-interface-derbi (Silent Spring Institute 2016).

Following this strategy, we arrived at a design for DERBI that supports both passive and proactive information seekers. By layering information throughout the report—a classic model for user interfaces (Shneiderman 1996)—users can navigate from a main summary page to more detailed information according to their interests. The summary page provides an overview of four major topics: “Chemicals We Found,” “Health Concerns,” “What You Can Do,” and “Overall Study Results.” These four topics are responsive to typical participant concerns in exposure biomonitoring (Brody et al. 2007) (Table 1). Personalized headlines—simple statements about noteworthy results—emphasize major findings for each participant. From the summary page, users can explore the report using navigation options embedded in the content or through a table of contents. Figure 1 illustrates key stages of the user experience. Within each section, participants can zoom in on chunks of related content, such as results for a particular chemical class or exposure reduction tips in a particular lifestyle area. Details-on-demand are accessible through “read more” buttons and lists of frequently asked questions. This information hierarchy builds users’ environmental health knowledge throughout the report, consistent with recent conceptualizations of stages of environmental health literacy (Finn and O’Fallon 2015).

**Table 1:** The Digital Exposure Report-Back Interface (DERBI) content relevant to typical participant questions.

Question	DERBI section	Responsive content
What did you find? How much? Is that high? Where did it come from?	Chemicals We Found	Personalized headlines highlight key exposure results defined by the research team. Strip plots show individual results for each chemical in relation to other study participants and a reference group. Text for a chemical group gives a brief history of use and potential sources of current exposure.
Is it safe?	Health Concerns	Groups of chemicals are affiliated with potential health effects, such as promoting cancer or affecting fertility. Language acknowledges uncertainty and warns against drawing a causal link between exposure results and illness.
How can I limit my exposure?	What You Can Do	Options for reducing exposure are organized by area, such as food or clothing, and address opportunities for (and limits on) individual and collective action.
What did you learn?	Overall Study Results	Text and graphs show what the research team has learned so far and provide broader public health context for individual results.
Note: Typical participant questions were first published in Brody et al. (2007).		

Using a model that has been successful in other studies, DERBI presents exposure results graphically using simple strip plots that many participants, of all levels of literacy and numeracy, are able to interpret (Figure 1). Graphs encode information visually, taking advantage of universal abilities to make higher than and lower than comparisons, so their interpretation relies less on formal education compared to reading text or tables. (Few 2004; Kosslyn 2006). For each analyte, the strip plot highlights the participant’s result in the context of the distribution of exposures in the study population. The plots also may show external benchmarks. For many biomonitored chemicals, human evidence of health effects is too limited to support the derivation of widely accepted safe or unsafe levels. As an alternative, population-level comparisons such as NHANES can provide a frame of reference for the study group. The strip plots provide intuitive information about how an individual compares to others and whether a result is “high” or “low.” Other graph formats might be more suitable for studies where chemicals have well-established guidelines for safety. Bar charts, for example, are easy to read (Haynes et al. 2016) and can demarcate whether a participant is above or below a guideline. Complementing the visual presentation of the data, a participant’s precise exposure level is displayed numerically next to the plot, and a complete table of results is also provided.

Reports are designed to be accessible to diverse participants. Unfamiliar terms that cannot be eliminated (e.g., units of measure) are defined, often in multiple contexts. The entire report underwent multiple rounds of one-on-one usability testing, with approximately 30 people testing versions of the GHS or CHDS prototypes. During usability testing, a researcher presents a prototype to an individual and asks them to think out loud and answer questions while navigating through the report. Prototypes were iteratively revised based on feedback from these sessions. Usability testers for the GHS report included convenience samples of university administrative staff and residents near the GHS public housing units, some of whom were non-native English speakers; testers of the CHDS prototype included convenience samples of middle-aged women demographically similar to CHDS participants. The CHDS report was also reviewed several times by the study’s Participant Advisory Council (Cresswell et al. 2012). Some elements of DERBI interface, including versions of the individual results graphs, were previously tested for other studies (Health Research for Action 2011; Judd and Plumb 2012). We continue to seek participant input to improve the report and refine our treatment of potentially confusing elements, such as the use of logarithmic scales.

### Scalable to Studies of All Sizes

DERBI’s digital platform enables rapid scaling of the user-centered experience to studies of any size: researcher effort per study is fixed no matter how many participants are enrolled. To accomplish this functionality, DERBI automatically generates individual results graphs and assigns personalized headlines to participants based on decision rules. Both manual and DERBI report-back require an initial time investment from researchers to determine a systematic protocol for personalizing text and graphs. In our experience, manually performing this personalization in a study of multiple chemicals can take up to an hour per participant. With DERBI, however, once programmed, the personalization protocol can be applied automatically across an unlimited number of reports, and it can be flexibly revised during report development with no additional time cost to implementation. DERBI also incorporates an electronic library of contextual information for participants, which is especially important to supporting small studies where producing interpretive content can be a greater barrier than personalizing reports.

**Figure 2:**
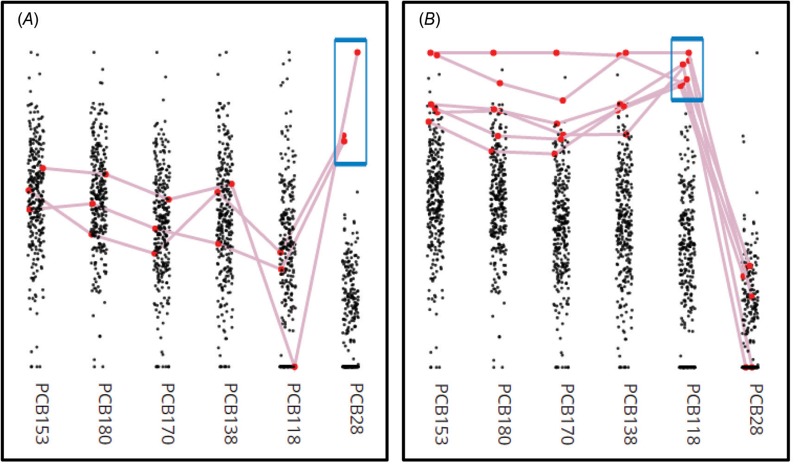
The Digital Exposure Report-Back Interface’s (DERBI) data visualization tool displays exposure distributions for the entire study and can help identify patterns. Clicking one point connects all the exposures belonging to a single participant in red, while dragging the blue box around multiple points dynamically highlights groups of participants. The vertical axis indicates concentration. In this example, showing optionally log-transformed and normalized data with non-detected values represented at zero, (*A*) participants highly exposed to PCB28 but not to more highly-chlorinated congeners likely have an exposure source in their home or workplace, such as old window caulk or electrical equipment, while (*B*) participants with the inverse pattern are likely primarily exposed to PCBs through their diet.

DERBI’s personalized headlines (Figure 1) are simple statements about a participant’s noteworthy results, based on rules specified by the researcher. For example, researchers can assign the headline “Your result was one of the (lowest/highest) for (chemical X)” to participants with exposures in a particular percentile range or in comparison to an external benchmark. A data visualization tool (Figure 2) showing study distributions can help researchers identify and author headlines for participants with unusual exposure profiles. Other sections also draw on these headlines. In the “What You Can Do” section, DERBI highlights exposure reduction tips related to a participant’s “higher” headlines. Researchers can limit how many headlines appear and specify how they are ordered. DERBI also can automatically order a participant’s results graphs by percentile rank or absolute concentration to draw attention to higher exposures. Automating the personalization of each report eliminates one of the most time-consuming tasks associated with report-back.

A library of reusable, modular content containing contextual information for interpreting personal results further reduces the burden of report-back for research teams. For each analyte or class of analytes, DERBI summarizes the exposure sources (including current and historical use), health effects, and exposure reduction tips, and addresses uncertainty in the evidence. Information about chemical sources and health effects was developed by R. Rudel and J. Brody and reviewed by principal investigators (G. Adamkiewicz, G. Chew, B. Cohn, R. Morello-Frosch) of studies using DERBI. It relies on fact sheets developed by Biomonitoring California, a state agency; chemical classifications by the International Agency for Research on Cancer and the U.S. EPA; determinations by the state of California under Proposition 65 (State of California 1986); and studies by the National Toxicology Program and in peer-reviewed literature. Summaries are written in lay language and reviewed for accuracy. Our goal is to convey the most salient health concerns for general population exposure levels—or for exposure levels specifically relevant to the study group—and offer the opportunity for precautionary action. To communicate knowledge gaps, we address uncertainty throughout the report, beginning in the informed consent. Reports state that the results are not diagnostic of health concerns (e.g., “You won’t be able to draw conclusions about the specific health implications for you or your family”). However, we are similarly straightforward about the information that does exist about the effects of these chemicals on cells, animals, and humans. Our approach aims to avoid both misplaced concern and false reassurance (Brody et al. 2007).

Chemical information modules currently include flame retardants, PFASs, pesticides, PCBs, phthalates, phenols, fragrance chemicals, and combustion byproducts. As we adapt DERBI for additional studies, the library will continue to grow. The library is currently maintained at the Silent Spring Institute in Massachusetts, but could become a collaborative online resource, although deciding how DERBI content will be moderated in the future remains an open question. Some content is available in Spanish and Chinese, as well as in English.

One major report element, the “Overall Study Results,” is unique to each study. Written by the study’s researchers, it summarizes the major contributions of the study to science and public health, and reminds participants of the broader context beyond their individual results. Study participants are often motivated by specific concerns about their community, such as the impact of nearby industrial polluters or a desire to address a particular disease (Brody et al. 2009; Ramirez-Andreotta et al. 2016). This section is a space where researchers can relate their findings to community-level concerns, keeping in mind that the results of greatest relevance to participants may differ from those of greatest scientific interest. To give participants their results promptly, reports may be prepared before the scientific findings of the overall study are known. However, participants are interested in and learn from descriptive information about exposures, and, in cohort studies, the overall results can draw on findings from earlier years. In the future, DERBI could iteratively return results as a study progresses.

Research teams can tailor other components of the report as needed. For example, scientists monitoring occupational cohorts might add exposure reduction tips for workplace conditions, and customizing pictures can make a report feel inviting to a particular audience. With computer programming experience, almost any element of the user interface can be modified.

The delivery method for report-back is best determined in conjunction with community input (Dunagan et al. 2013; Haynes et al. 2016). Reports can be delivered in-person by study personnel, or by mail or web. Regardless, all reports give contact information for the research team, and in studies that have reported personal results using DERBI or other methods, some participants did use the contact information to call the study team with questions from their reports. An advantage of web reports is that they can include extensive information without looking many-pages long, because participants navigate to information layers without seeing everything at once. However, print reports are advisable for communities with limited internet access. Currently, web reports are optimized for a personal computer or tablet, but developing a mobile interface is a priority to provide better access in low-income communities (Smith 2015). Web reports are hosted on a secure server, and no reports—print or web—contain personally identifiable information.

### Research Tools for Data Exploration and Collection

DERBI incorporates analytical tools that we developed to aid our own efforts interpreting and reporting exposure results. To facilitate researchers’ ability to examine the full range of a study’s exposure distributions, DERBI displays biomonitoring data sets using parallel coordinate plots (Figure 2). The plots help researchers identify patterns, and a highlighting tool allows researchers to examine individual exposure profiles across multiple chemicals. Taken together with other environmental health expertise, these visualizations may suggest particular exposure sources or behaviors, as well as provide insight on product formulations (Figure 2). Researchers can relay these insights to participants by assigning them unique headlines. We anticipate that report-back will stimulate researchers to consider the questions—and intervention opportunities—posed by high exposures and common chemical mixtures.

Web-based reports support additional tools for learning about, and enhancing, participants’ experience. Using web analytics (with participants’ consent), researchers can track which participants have opened their reports and learn about participant behavior, such as how long they spent on the site and which paths they took through the report. In addition to passive data collection, web-based reports offer opportunities for active engagement with users, such as integrated evaluation tools (pre- and post-tests) or more sophisticated educational interventions. In usability tests, participants often seek validation that they are interpreting their results correctly, and the web platform has the potential to offer a “guided” or “tutorial” experience by incorporating interactive activities that provide immediate feedback.

### User Feedback

We conducted semi-structured interviews with 22 participants who received personal reports in CHDS and systematically coded responses using qualitative analysis methods. Using a mental models approach, 20 interviews are considered sufficient to capture the range of perspectives and structure of thinking about a problem, though not to reliably quantify the frequency of occurrence in the population (Morgan et al. 2002). Out of 20 participants who answered the question, “Did you find the report helpful,” 18 (90 percent) responded yes. Eight participants cited exposure reduction as a reason that the report was helpful, for example, “It’s good to know things that are harming my body so that I can make changes to the way I live my life.” Seven participants stated the reports improved their general knowledge or awareness of chemical exposures: “I guess just giving me a better understanding about the air, chemicals, stuff that I use around the house or at home, stuff that I inhale.”

Out of 18 participants who answered the question, “What did you think about the way the results were presented,” 15 (83%) responded positively. Seven volunteered that the results were clear or easy to understand. Four mentioned that they liked being able to compare their result to the study distribution and to national averages: “I liked the way they did that. It showed where I was at and where other people were at.” The three participants with negative responses had difficulty understanding the graphs or requested additional information to help them interpret the results.

### Limitations

DERBI assists, but does not replace, the role of researchers in conducting report-back. Even with DERBI’s digital framework, researchers must still devote effort to write study-specific content, tailor reports to community context, and update the digital content library. As more studies adopt DERBI, it is our hope that participating researchers will contribute to library maintenance and growth, so that no single study team is highly burdened. Additional research about baseline environmental health knowledge in diverse communities will also help researchers prepare responsive reports.

Knowledge of the health effects of emerging contaminants is evolving and sometimes contested, and DERBI’s content, while curated by environmental health scientists, is necessarily limited by what is known. However, the intention of report-back for chemicals with uncertain health effects is to allow participants to make informed decisions about precautionary action, while recognizing that public health recommendations can change as new evidence emerges. Providing participants with knowledge, including simple options for reducing chemical exposures, allows participants to decide independently whether to take action based on their individual values related to risk. Our approach is similar to community outreach in NIEHS-supported studies such as the Breast Cancer and the Environment Research Program, Children’s Environmental Health Centers, and Superfund Research Program, which disseminate fact sheets that include tips for reducing exposures, including for chemicals without established health guidelines (e.g., BCERP 2013).

### Future Directions

We are currently adapting DERBI for four more studies and conducting detailed analysis of participants’ experience receiving DERBI web reports in CHDS. In the future, we hope to provide a user-centered interface for other researchers to create DERBI reports for their studies, share new content, and discuss experiences. We also plan to develop a smartphone interface for participants who rely on a mobile device for internet access. One goal of report-back is to help participants understand the connections between environmental exposures and health, so future research can focus on evaluating how report-back affects environmental health literacy—which encompasses both the knowledge and the self-efficacy needed to understand, assess, and make decisions about exposures (Finn and O’Fallon 2015). As part of our research, we plan to test approaches specifically designed to improve user confidence in interpreting their report.

## Conclusions

Digital methods have the potential to revolutionize report-back by removing barriers to the production of high-quality, personalized reports. We used these methods to establish DERBI prototype, including a user-centered template for results return, automated rules to personalize reports, a digital repository for content, interactive graphs for data exploration, and built-in analytics to study participant behavior. User feedback showed that many participants understood their results and found the report helpful. Given its flexibility and efficiency, we posit that DERBI can facilitate report-back both in very large studies, potentially including NHANES, where the number of participants would otherwise make personalization difficult, and in small studies with limited resources. For the future, DERBI has the potential to contribute to the critical task of integrating individual-level environmental exposures into precision medicine, so that genomic and environmental data are collected—and returned—in tandem. Digital report-back can also extend to self-tracking by personal sensors or software applications. Digital methods such as DERBI that facilitate report-back help researchers meet ethical obligations by removing practical barriers, encourage study participation by opening communication, and discover insights about their data. Most importantly, DERBI can stimulate environmental health learning and provide participants with knowledge they can use to take individual and collective action to reduce harmful exposures.

## References

[r1] Adams C Brown P Morello-Frosch R Brody JG Rudel R Zota A 2011 Disentangling the exposure experience: the roles of community context and report-back of environmental exposure data. J Health Soc Behav 52 2 180 196, doi:10.1177/0022146510395593 21673146 PMC3175404

[r2] Altman RG, Morello-Frosch R, Brody JG, Rudel R, Brown P, Averick M (2008). Pollution comes home and gets personal: women’s experience of household chemical exposure.. J Health Soc Behav.

[r3] Barlow J, Kushi L (2011). Communicating Individual-Level Results to Participant Families. Breast Cancer and the Environment Research Program Annual Meeting, 16-18 Nov 2011, Cincinnati, OH.. http://www.bcerp.org/2011mtg/14.panel.pdf.

[r4] BCERP (Breast Cancer and the Environment Research Program) (2013). Your daughter and breast cancer. Reducing her risk now.. http://info.bcerp.org/images/docs/1386_NIEHS_BCERP_Mother_Toolkit_Final_PC.pdf.

[r5] Becker K, Seiwert M, Casteleyn L, Joas R, Joas A, Biot P (2014). A systematic approach for designing a HBM pilot study for Europe.. Int J Hyg Environ Health.

[r6] Bennett D Bellinger DC Birnbaum LS Bradman A Chen A Cory-Slechta DA 2016 Project TENDR: targeting environmental neuro-developmental risks: the TENDR Consensus Statement. Environ Health Perspect 124 7 A118 A122, doi:10.1289/EHP358 27479987 PMC4937840

[r7] Brody JG Dunagan SC Morello-Frosch R Brown P Patton S Rudel RA 2014 Reporting individual results for biomonitoring and environmental exposures: lessons learned from environmental communication case studies. Environ Health 13 40, doi:10.1186/1476-069X-13-40 24886515 PMC4098947

[r8] Brody JG, Morello-Frosch R, Brown P, Rudel RA, Altman RG, Frye M (2007). Improving disclosure and consent: “Is it safe?”: new ethics for reporting personal exposures to environmental chemicals.. Am J Public Health.

[r9] Brody JG, Morello-Frosch R, Zota A, Brown P, Pérez C, Rudel RA (2009). Linking exposure assessment science with policy objectives for environmental justice and breast cancer advocacy: the Northern California Household Exposure Study.. Am J Public Health.

[r10] CDC (Centers for Disease Control and Prevention) (2015). 2015 NHANES Health Measurements.. https://www.cdc.gov/nchs/data/nhanes/nhanes_15_16/2015_Health_Measurements_List.pdf.

[r11] CDC (2016). What Do Parents Need to Know to Protect Their Children?. https://www.cdc.gov/nceh/lead/acclpp/blood_lead_levels.htm.

[r12] CHDS (Child Health and Development Studies) (2016). About Us.. http://www.chdstudies.org/about_us/index.php.

[r13] CMS, CDC, OCR (Centers for Medicare & Medicaid Services, Centers for Disease Control and Prevention, Office for Civil Rights) (2014). CLIA Program and HIPAA Privacy Rule; Patients’ Access to Test Reports. Final Rule. Fed Regist 79(25):7289–7316.. https://www.gpo.gov/fdsys/pkg/FR-2014-02-06/pdf/2014-02280.pdf.

[r14] Coombs KC Chew GL Schaffer C Ryan PH Brokamp C Grinshpun SA 2016 Indoor air quality in green-renovated vs. non-green low-income homes of children living in a temperate region of US (Ohio). Sci Total Environ 554–555 178 185, doi:10.1016/j.scitotenv.2016.02.136 PMC4818700 26950631

[r15] Cresswell S, Plumb M, Cohn B, Cirillo P (2012). From “Research On” to “Research With” Cohort Members: A Case Study. 140th Annual Meeting of the American Public Health Association, 27-31 October 2012, San Francisco, CA.. https://apha.confex.com/apha/140am/webprogram/Paper258548.html.

[r16] Day B, Langlois R, Tremblay M, Knoppers BM (2007). Canadian Health Measures Survey: ethical, legal and social issues.. Health Rep.

[r17] Di Renzo GC Conry JA Blake J DeFrancesco MS DeNicola N Martin JN 2015 International Federation of Gynecology and Obstetrics opinion on reproductive health impacts of exposure to toxic environmental chemicals. Int J Gynaecol Obstet 131 3 219 225, doi:10.1016/j.ijgo.2015.09.002 26433469 PMC6663094

[r18] Dunagan S, Brody JG, Morello-Frosch R, Brown P, Goho S, Tovar J, et al (2013). When Pollution is Personal. Handbook for Reporting Results to Participants in Biomonitoring and Personal Exposure Studies.. http://www.silentspring.org/sites/default/files/personal_exposure_report_handbook_0.pdf.

[r19] Esch T Mejilla R Anselmo M Podtschaske B Delbanco T Walker J 2016 Engaging patients through open notes: an evaluation using mixed methods. BMJ Open 6 1 e010034, doi:10.1136/bmjopen-2015-010034 PMC4735137 26826154

[r20] Few S (2004). *Show Me the Numbers: Designing Tables and Graphs to Enlighten*..

[r21] Finn S O’Fallon L 2015 The emergence of environmental health literacy—from its roots to its future potential. Environ Health Perspect, doi:10.1289/ehp.1409337 PMC5382009 26126293

[r22] Gore AC Chappell VA Fenton SE Flaws JA Nadal A Prins GS 2015 EDC-2: the Endocrine Society’s second scientific statement on endocrine-disrupting chemicals. Endocr Rev 36 6 E1 E150, doi:10.1210/er.2015-1010 26544531 PMC4702494

[r23] Haynes EN Elam S Burns R Spencer A Yancey E Kuhnell P 2016 Community engagement and data disclosure in environmental health research. Environ Health Perspect 124 2 A24 A27, doi:10.1289/ehp.1510411 26829152 PMC4749085

[r24] Health Research for Action (2011). Chemicals In Our Bodies Project: Report on Usability Tests of Report-back Materials (English and Spanish)..

[r25] Hernick AD Brown MK Pinney SM Biro FM Ball KM Bornschein RL 2011 Sharing unexpected biomarker results with study participants. Environ Health Perspect 119 1 1 5, doi:10.1289/ehp.1001988 PMC3018486 20876037

[r26] Holtzblatt K (2009). Contextual design. In Human-Computer Interaction: Development Process. A. Sears A, Jacko J, eds..

[r27] Hood L Auffray C 2013 Participatory medicine: a driving force for revolutionizing healthcare. Genome Med 5 110, doi:10.1186/gm514 24360023 PMC3978637

[r28] Judd S, Plumb M (2012). *Reporting Personal Exposures to Environmental Chemicals: Focus Group Feedback*.. Child Health and Development Studies, Public Health Institute, Oakland.

[r29] Judge JM Brown P Brody JG Ryan S 2016 The exposure experience: Ohio River Valley residents respond to local perfluorooctanoic acid (PFOA) contamination. J Health Soc Behav 57 3 333 350, doi:10.1177/0022146516661595 27601409

[r30] Kosslyn SM (2006). *Graph Design for the Eye and Mind*..

[r31] Minkler M, Wallerstein N, eds (2008). *Community-Based Participatory Research for Health: From Process to Outcomes*..

[r32] Morello-Frosch R Brody JG Brown P Altman RG Rudel RA Pérez C 2009 Toxic ignorance and right-to-know in biomonitoring results communication: a survey of scientists and study participants. Environ Health 8 6, doi:10.1186/1476-069X-8-6 19250551 PMC2654440

[r33] Morello-Frosch R Varshavsky J Liboiron M Brown P Brody JG 2015 Communicating results in post-Belmont era biomonitoring studies: lessons from genetics and neuroimaging research. Environ Res 136 363 372, doi:10.1016/j.envres.2014.10.001 25460657 PMC4262542

[r34] Morgan MG, Fischhoff B, Bostrom, A, Atman CJ (2002). *Risk Communication: A Mental Models Approach*..

[r35] Nelson JW Scammell MK Altman RG Webster TF Ozonoff DM 2009 A new spin on research translation: the Boston Consensus Conference on Human Biomonitoring. Environ Health Perspect 117 4 495 499, doi:10.1289/ehp.0800037 19440485 PMC2679590

[r36] NRC (National Research Council) (2006). *Human Biomonitoring for Environmental Chemicals*..

[r37] Quandt SA, Doran AM, Rao P, Hoppin JA, Snively BM, Arcury TA (2004). Reporting pesticide assessment results to farmworker families: development, implementation, and evaluation of a risk communication strategy.. Environ Health Perspect.

[r38] Ramirez-Andreotta MD Brody JG Lothrop N Loh M Beamer PI Brown P 2016 Reporting back environmental exposure data and free choice learning. Environ Health 15 2, doi:10.1186/s12940-015-0080-1 26748908 PMC4707004

[r39] Shneiderman B (1996). The Eyes Have It: A Task by Data Type Taxonomy for Information Visualizations.. Proceedings: IEEE Symposium on Visual Languages, 3–6 September 1996, Boulder, Colorado.

[r40] Silent Spring Institute (2016). Digital Exposure Report-Back Interface (DERBI).. http://silentspring.org/derbi.

[r41] Smith A (2015). U.S. Smartphone Use in 2015.. http://www.pewinternet.org/2015/04/01/us-smartphone-use-in-2015/.

[r42] State of California (1986). Safe Drinking Water and Toxic Enforcement Act of 1986.. California Health and Safety Code §§ 25249.5-25249.13 (November 4, 1986).

[r43] State of California (2006). Senate Bill No. 1379, Chapter 599, California Environmental Contaminant Biomonitoring Program.. California Health and Safety Code ßß 105440–105444 (September 29, 2006).

[r44] The White House (2015). Precision Medicine Initiative: Privacy and Trust Principles.. https://www.whitehouse.gov/sites/default/files/microsites/finalpmiprivacyandtrustprinciples.pdf.

[r45] UNEP/WHO (United Nations Environment Programme, World Health Organization) (2013). State of the Science of Endocrine Disrupting Chemicals 2012. Bergman A, Heindel JJ, Jobling S, Kidd KA, Zoeller RT, eds. 2013.. http://unep.org/pdf/9789241505031_eng.pdf.

[r46] U.S. EPA (U.S. Environmental Protection Agency) (2008). Scientific and Ethical Approaches for Observational Exposure Studies. EPA/600/R-08/062. Research Triangle Park:U.S. EPA.. https://cfpub.epa.gov/si/si_public_record_report.cfm?dirEntryId=191443.

[r47] Wolf SM 2013 Return of individual research results and incidental findings: facing the challenges of translational science. Annu Rev Genomics Hum Genet 14 557 577, doi:10.1146/annurev-genom-091212-153506 23875796 PMC4452115

[r48] Wu N McClean MD Brown P Aschengrau A Webster TF 2009 Participant experiences in a breastmilk biomonitoring study: a qualitative assessment. Environ Health 8 4, doi:10.1186/1476-069X-8-4 19226469 PMC2649062

